# Strengthening expertise for health technology assessment and priority-setting in Africa

**DOI:** 10.1080/16549716.2017.1370194

**Published:** 2017-10-16

**Authors:** Jane E Doherty, Thomas Wilkinson, Ijeoma Edoka, Karen Hofman

**Affiliations:** ^a^ School of Public Health, Faculty of Health Sciences, University of the Witwatersrand, Parktown, Johannesburg, South Africa; ^b^ PRICELESS SA (Priority Cost-Effective Lessons for Systems Strengthening South Africa), School of Public Health, Faculty of Health Sciences, University of the Witwatersrand, Parktown, Johannesburg, South Africa

**Keywords:** Research, capacity, networks, health technology assessment

## Abstract

**Background**: Achieving sustainable universal health coverage depends partly on fair priority-setting processes that ensure countries spend scarce resources wisely. While general health economics capacity-strengthening initiatives exist in Africa, less attention has been paid to developing the capacity of individuals, institutions and networks to apply economic evaluation in support of health technology assessment and effective priority-setting.

**Objective**: On the basis of international  lessons, to identify how research organisations and partnerships could contribute to capacity strengthening for health technology assessment and priority-setting in Africa.

**Methods**: A rapid scan was conducted of international formal and grey literature and lessons extracted from the deliberations of two international and regional workshops relating to capacity-building for health technology assessment. ‘Capacity’ was defined in broad terms, including a conducive political environment, strong public institutional capacity to drive priority-setting, effective networking between experts, strong research organisations and skilled researchers.

**Results**: Effective priority-setting requires more than high quality economic research. Researchers have to engage with an array of stakeholders, network closely other research organisations, build partnerships with different levels of government and train the future generation of researchers and policy-makers. In low- and middle-income countries where there are seldom government units or agencies dedicated to health technology assessment, they also have to support the development of an effective priority-setting process that is sensitive to societal and government needs and priorities.

**Conclusions**: Research organisations have an important role to play in contributing to the development of health technology assessment and priority-setting capacity. In Africa, where there are resource and capacity challenges, effective partnerships between local and international researchers, and with key government stakeholders, can leverage existing skills and knowledge to generate a critical mass of individuals and institutions. These would help to meet the priority-setting needs of African countries and contribute to sustainable universal health coverage.

## Background

This article reflects on the international experience of building capacity for setting health system priorities in low- and middle-income countries, with a particular focus on Health Technology Assessment (HTA) and how research organisations can contribute to capacity development.

HTA is one tool that supports the formulation of health policies that decide on the allocation of resources between different activities. Using multidisciplinary analyses, it examines the health, economic, social and ethical implications of the use of new (and existing) ‘technologies,’^1^ broadly defined as any interventions to improve health, including public health interventions. These analyses are typically expressed as a comparison between the costs and impact of an intervention compared to an identified comparator (where the impact may be expressed as the delivery of a unit of service, a health outcome or the monetary value that society accords this outcome). Choices between different interventions can then be made on the basis on how much value for money they are able to offer, and the extent to which they address the major causes of the burden of disease and other factors that may be of interest to the decision maker.

HTAs consequently can inform the development of clinical guidelines, the composition of benefit packages and reimbursement mechanisms for health care providers, and are essential to the realisation of Universal Health Coverage policies [1].

The clinical and economic assessment of interventions is the particular concern of HTA, given that resources are insufficient to cope with the existing burden of disease. Economic analyses are therefore the core activity of HTA. However, to have policy impact, the findings of these evaluations need to be inserted into government’s priority-setting processes, where they are weighed against numerous other concerns. Participation in certain stages of these processes is also a function of HTA practitioners and therefore this article interprets HTA as comprising the functions of both economic evaluation and other components of priority-setting, as described later.

The specific contribution of this article is therefore to extend the existing literature on building capacity for health economics research in low- and middle-income countries, to the specific area of economic evaluation in support of priority-setting, and hence policy-making. In addition, the article seeks to provide practical advice to researchers and research organisations working in Africa on how they could contribute to the strengthening of national priority-setting processes, in the absence of substantial capacity within most African governments to do so internally.

## Methods

This article draws on a rapid scan of three complementary sources to collect international experience on HTA capacity development in Africa [2]. First, a scan was conducted in 2015 of the published and grey literature from low- and middle-income countries on building capacity in health systems and policy research (with a focus on health economics and HTA). Search terms included ‘capacity building,’ ‘health/health systems research,’ ‘health economics,’ and ‘health technology assessment,’ and 44 relevant documents were identified.

Second, experiential lessons were contributed by presentations and discussions from two side meetings on HTA at the Prince Mahidol Award Conference in Bangkok, Thailand, in January 2015, each of which consisted of at least 30 participants.^2^

Third, experiential lessons were also contributed by the deliberations of a national workshop on HTA run by PRICELESS SA (Priority Cost Effective Lessons for Systems Strengthening in South Africa) at the Wits School of Public Health, in Johannesburg, South Africa, in March 2015.^3^ This workshop also consisted of at least 30 participants.

These sources were analysed according to themes. Triangulation of data was used to identify themes and lessons that were consistent across several sources.

The analysis was guided by a conceptual framework for understanding organisational capacity that sees capacity as not only the numbers and skills of individuals in an organisation, but also the strengths of the organisation itself, the extent to which the organisation is networked with other relevant actors in performing its tasks, the positive features of the public sector environment in which the organisation has to operate, and a supportive contextual environment [3].

The article first presents international lessons in successful capacity-building in health economics more generally, and economic evaluation and health technology assessment more specifically, according to this framework. The authors then briefly assess the various dimensions of capacity constraints in Africa, based on their collective experience, and then formulate recommendations as concrete steps that research organisations could take to contribute towards HTA capacity-development in Africa, recognising that these are tentative and highly dependent on the contextual factors pertaining in each country.

One limitation of this approach is that there are relatively few sources dealing with the focus of this article and they vary in format and the level of detail they present. They also come from contexts that are not necessarily comparable. Another limitation is that the workshop sources are not necessarily representative and there is a risk that the authors were selective in recording the discussions in these workshops. In the absence of better guidance, the opinions of the authors of necessity influenced the generation of recommendations heavily. As a consequence, the recommendations are presented as tentative, with the intention of prompting debate. Nonetheless, the authors feel that, although based on a rapid scan of such sources, the article draws on the ‘tacit knowledge’ of a wide range of experts with deep experience of implementing both HTA and broader priority-setting processes, and that this experience provides fairly consistent guidance.

## Results

As described earlier, this section presents lessons grouped according to the dimensions of capacity: a supportive environmental context (in this case, the presence of political support); a supportive public sector institutional environment (in this case, the presence of a formal and effective priority-setting process); effective networks of HTA experts; strong HTA research institutions; and skilled HTA staff.

### Building political support for HTA

A supportive environmental context makes it easier for organisations to build capacity, both internally and externally [3]. Political will is one element of a supportive context and especially important for effective HTA systems [4]. Internationally, HTA has begun to be integrated closely into government decision-making in some countries (for example, in England and Thailand). This has come with greater awareness of the need to consider not just efficiency concerns but also issues of equity and other social values, whilst balancing the demands of different stakeholders [4–6]. At the same time, the methodologies underlying HTA have become more sophisticated, taking into account both delivery platforms and the synergies between different interventions.

The impetus internationally towards universal health coverage (especially in largely publicly financed health systems) has helped to spur these developments [7,8]: this is because decisions about the definition of affordable benefit packages, purchase prices for pharmaceuticals and reimbursement rates for providers can be better informed by economic analyses. It also ensures that opportunity costs are considered. Further, universal health coverage creates a climate in which it is possible to build popular support for fair and efficient priority-setting.

### Building public sector institutional capacity for HTA

Public sector institutions need to be able to drive and support effective priority-setting processes. The international literature identifies the necessary components of a fair and sound priority-setting process that is based on HTA, and effectively manages political, commercial, advocacy and donor interests (Box 1).

Such an HTA process could adjudicate different approaches to public health interventions, just as it does, more commonly, for pharmaceuticals, medical equipment and procedures. It needs to be formalised, transparent, robust and protected from political and commercial pressures. It also needs to be funded sustainably (for example, through government grants and not only ad hoc research grants) and should avoid funding from stakeholders with vested interests. It requires considerable expertise within government to lead and manage, not only in terms of technical analysis but also in terms of stakeholder management. The literature from South-East Asia accordingly emphasises the importance of developing a core HTA team (or agency) to take on these tasks. Successful HTA agencies in this region have as many as 20 to 80 full-time academic staff [9].

The HTA unit or agency does not necessarily have to conduct all economic analyses itself: it could commission research from a variety of external institutions as well (which is the model followed by Thailand, for example), or the broad majority of research could be commissioned and conducted by academic centres externally (which is the model at the National Institute for Health and Care Excellence (NICE) in England). External commissioning requires strict guidelines for the conduct, reporting and implementation of such research. Importantly, while an HTA unit or agency is responsible, as a neutral arbitrator, for putting forward recommendations, it is not always responsible for deciding whether these recommendations should be incorporated into policy.

Analyses of a range of low- and middle-income countries that have been less successful in ensuring robust HTA processes, identified a number of challenges [5] including: a shortage of local research capacity; weaknesses in legal and institutional structures; a shortage of quality data; flawed HTAs as a result of poor research methods, especially where there are no standardised HTA research guidelines and processes; research seldom directed towards major health problems; benefit packages not updated on the basis of new data and products; minimal involvement of stakeholders; budget considerations not taken into account; and HTA evidence not used appropriately because health professionals and policy-makers do not have a good understanding of HTA and/or research is not timely.

### Successful networking between HTA experts

This section focuses on the capacity of the ‘task network’ of HTA experts in government, research organisations, consultancies and private health financing organisations that need to be linked effectively in order to identify priority areas of research, collaborate on developing methodologies or conducting research, and discuss the implications of research findings.

The benefits of regional and international research linkages are emphasised by studies from Kenya, Malawi, Nigeria and Thailand, amongst others [9–13]. Experience from South Africa shows that the capacity of relatively small health economics research units to take on large projects was made possible through partnering with like-minded organisations [14,15]. This created a critical mass to undertake work, promoted the sharing of methods between the partners (thereby building capacity) and enhanced the profile of the research partners (thereby extending their policy influence).

With respect to international lessons for successful networking, it is clear that networks require very active management and careful negotiation of the responsibilities of, and relationships between, the different partners (Box 2).

### Strengthening the organisational capacity of HTA research groups

When it comes to building research capacity, the focus is often on individual researchers. However, to ensure that research capacity is enduring, and not overly dependent on a few remarkable researchers, it is critical to strengthen the organisational capacity of research institutions: this tends to be a relatively neglected component of donor-funded capacity-building efforts [16]. This section therefore looks at the international experience of building the organisational capacity of research groups, including how to optimise the support provided by international partners.

Experience from Africa and South-East Asia identifies multiple challenges to building research capacity [17,18]. These include inadequate financial and human resources, problems retaining skilled staff, as well as weak governance and management. Capacity-building partnerships with international partners often struggle with finding a compromise between their research and methodological interests and priorities of local and international researchers, imbalances in power between research partners, and a lack of trust. This notwithstanding, there are some examples of successful partnering between Northern and Southern partners in health economics capacity-building in Africa (such as the Health Economics and Policy Network (HEPNet) [14,19,20].

An interesting point is that Mexican and Thai researchers found that the strong organisational capacity of their research groups was partly accounted for by their frequent face-to-face interactions with policy-makers (in meetings and on training programmes) [21,22]. Their trustworthiness was based on the relevance of their work to contemporary issues, long-term innovative research programmes that provided timely information, high quality research underpinned by local and international peer review, core values consistent with the social objectives of government, intellectual independence, being uncompromising in avoiding research funding from organisations with vested interests, and transparency. Studies from South Africa also highlight the important synergy between policy-makers and researchers in training programmes [14,23]: while researchers are able to disseminate their research findings, policy-makers are able to convey research priorities to researchers as well as implementation challenges.

All these international studies confirm the need to ensure core funding for research groups, which are typically grant-funded. This puts a heavy burden of continual fund-raising on the shoulders of senior staff, makes it difficult to attract high calibre staff because of the lack of job security, forces research units to cross-fund important activities such as capacity-building from research funds, and distorts research agendas according to the needs of international funders.

Thus, one of the important factors that has contributed to the success of the Thai Health Intervention and Technology Assessment Programme has been substantial funding by government and international agencies, although this has brought its own challenges as the Programme has had to carefully manage its academic independence [11,22]. Core government funding also provided stability and opportunities for expansion of health systems research in China and Mexico which have seen growth in the number of researchers, and an improved ability to take on long term programmes of research that have a direct and profound influence on government reforms, whilst retaining some degree of academic independence [21,24].

### Building the capacity of individual research staff to conduct and apply HTAs

Local training programmes were an important explanatory precursor to HTA agencies in Korea, Malaysia, Taiwan and Thailand [9]. In these countries, training programmes included short courses and workshops as well as Master’s and PhD degrees. PhD training is critical to provide the advanced skills required to conduct complex research and lead research teams [25], which are often requirements for advanced HTAs.

With respect to where researchers could access training, there is a range of opportunities for regional or international training. A tactic used by the Thai government was to give its staff the opportunity to undergo PhD training at the London School of Hygiene and Tropical Medicine by providing bursaries [11]. The main gap in this sort of training, though, is the lack of local technical support that allows close and on-going supervision of local research studies. Further, formal training by international experts is expensive, especially when located overseas: it is therefore not a sustainable or complete alternative to local training. Nonetheless, informal exchanges with overseas partners remain very useful. A novel initiative focussed on the specific needs of advanced PhD training in Africa, and which is based in Africa and allows collaboration and learning across countries, is the Consortium for Advanced Research Training in Africa (CARTA) programme [25].

Another dimension of capacity-building is building the capacity of staff working internally in a unit. This is an important component of any research capacity-building strategy in African countries, as they struggle with a chronic shortage of mid-level research staff. In South Africa, this seems to be because, as soon as staff gain sufficient skills within a research environment they become easily employable in the government and private sectors [14,15,23]. This results in a severely over-stretched cohort of senior researchers, which in turn jeopardises the sustainability of training programmes relying on their expertise and guidance.

Lastly, a particular complexity of HTA is that it requires multi-disciplinary skills: researchers from specific disciplinary backgrounds may need to learn and apply skills from other disciplines or learn how to work effectively in multi-disciplinary teams.

## Discussion and recommendations

This section discusses the lessons from international experience presented above and generates options for action by African countries, recognising that countries’ choices will depend on their own particular contexts and current capacities. These options have been developed on the basis of the authors’ assessment of prevailing conditions in Africa, the deliberations of workshops, and advice from international colleagues, and are hence tentative, requiring further debate. The recommendations are presented according to the same dimensions of capacity as used in the previous section (see Table 1).

**Table 1.** Summary of recommendations for researchers

Component of capacity-building	Recommendations
Building political support for HTA	Develop support among politicians and in broader society Develop materials that explain the concept of HTA and demonstrate its application Use occasions such as the meetings of HTA and related societies to disseminate information, provide training and garner ideas
Building public sector institutional capacity for HTA	Develop a good relationship with key individuals in the Ministry of Health and Treasury Develop a better understanding of current priority-setting structures and processes in government Identify a few concrete, useful and immediate interventions to enhance government capacity Undertake a collaborative, demonstration research project Collaborate with the Ministry of Health on facilitating the development of official guidelines for HTA and a threshold for cost-effectiveness Discuss with the Ministry of Health possibilities for the longer-term training of key individuals earmarked to develop in-house HTA expertise Explore how research groups could help the Ministry of Health set up an HTA unit in the longer term
Successful networking between HTA experts	Invest in expanding relationships with key stakeholders Consider partnering with one or two local organisations on a concrete project Explore what role the private sector could play in supporting the development of an HTA system Consider establishing a formal regional network
Strengthening the organisational capacity of HTA research groups	Actively manage the needs and concerns of the various partners in research partnerships Incorporate a longer-term funding strategy into an organisational capacity-building plan
Building the capacity of individual staff to conduct and apply HTA research	Survey key stakeholders regarding training needs for HTA Seek funding and technical support for designing appropriate courses Develop a strategy for recruiting local researchers into the organisation, and developing the capacity of all internal staff

### Building political support for HTA

The current international focus on Universal Health Coverage is likely to create an increased demand by governments for HTA, especially given current economic constraints. This positive external environment is tempered by a number of challenges in Africa. These include: limited capacity in health economics; the absence of formally established HTA agencies in African Ministries of Health to coordinate the HTA process (unlike in several South-East Asian countries); and decision-making processes that are often fragmented across tiers of government and between ‘silos’ within the Ministry of Health.

*Recommendations to research institutions seeking to build political support for HTA in Africa*

1. See the development of political support as a critical aspect of capacity building and develop a detailed understanding of how to stimulate and sustain this support. While government is a key stakeholder in this regard, and should undoubtedly be the focus of initial efforts, it is also important to build support for HTA-informed decision-making in broader society (including health care practitioners, civil society groups, patient groups and the media). This is because of the highly contested nature of resource allocation policy.
2. As HTA is a complex and sometimes alienating concept (both because what the word ‘technology’ encompasses is not immediately clear, and because the techniques HTA uses are highly specialised and technical), develop simple and eye-catching materials that explain the concept and demonstrate its application.^4^ Thus, show how, internationally (and specifically in low- and middle-income countries), HTA has contributed to affordable and equitable health care and has led to improved health outcomes. Also, provide concrete examples of how HTA could be incorporated into decision-making.
3. Use occasions such as the meetings of HTA and related societies to disseminate information on the practical use of HTA evidence, provide training to policy-makers and managers on how to commission and use evidence, and garner ideas from policy-makers and managers on how best to support their decision-making needs.

### Building public sector institutional capacity for HTA

One of the motivations behind this article is an understanding that public sector institutional capacity in Africa as a whole is currently relatively weak with respect to HTA. As discussed earlier, international experience shows that it can take a long time to develop an HTA unit capable of functioning at a high level (in Thailand it took over two decades of experimenting with the creation of various units and programmes before the institutionalisation of a national agency in 2007, for example).

An important component of the development of national HTA capacity is the publication of national guidelines for the conduct of HTA. Only a limited number of protocols have been developed for the African context and the appropriateness of their design and application still needs to be investigated further. The International DecisionSupport Initiative’s Reference Case for Economic Evaluation provides a principle-based framework for standardisation of methods and was specifically developed for use in low- and middle-income settings.^5^

*Recommendations to research institutions seeking to build the capacity of Ministries of Health and Treasuries to commission and use HTA evidence*

1. Develop a good relationship with key individuals in the Ministry of Health and Treasury, seeking their opinions on what sort of support they require to build awareness on how to use HTA evidence, and initiate, structure and facilitate a process that supports more rational commissioning and use of HTA on a national scale. In particular, develop an understanding of how to progress beyond in-principle support to effective processes that gradually improve the way that HTA evidence is incorporated in government decision-making.
2. Develop a better understanding of current priority-setting structures and processes in government (at national, provincial, district and facility levels) as well as key flaws in these processes and capacity constraints.
3. Identify a few concrete, useful and immediate interventions to enhance government capacity, not just for understanding, conducting or commissioning HTAs, but also on how to manage what can become highly charged political processes. This could include, for example, such activities as coaching committee members on what is expected of a priority-setting committee, how to interrogate evidence and arranging policy dialogues on pertinent issues and advising lower levels of the health system on how to implement HTA-related advice. These could be done at a national level or, in a decentralised system, at lower levels. They would be a mechanism to give policy-makers a concrete understanding of what is possible with HTA, develop trust between policy-makers and technical experts, and give technical experts a better understanding of the context facing decision-makers.
4. Undertake a demonstration research project, possibly in collaboration with other country research partners, in order to provide the Ministry of Health with a ‘small win’ and in the process provide capacity-strengthening opportunities to government and academic staff.
5. Explore the possibility of collaborating with the Ministry of Health on facilitating the development of official guidelines for HTA and conducting research to estimate a locally-appropriate cost-effectiveness threshold (this could become a collaborative activity involving a number of research groups or associations). An analysis of what data are available and mechanisms for dealing with data weaknesses could form part of this research agenda.
6. Discuss with the Ministry of Health possibilities for the longer-term training of key individuals earmarked to develop in-house HTA expertise (for example, mentoring, short courses and internships at research organisations).
7. Explore whether and how, in the longer term, research groups could help the Ministry of Health perform HTA functions with the intention of eventually graduating towards a fully-fledged HTA unit. Such functions could include convening meetings to identify research priorities and to commission research.

### Successful networking between HTA experts

Africa has produced some cost-effectiveness-related studies and has some well-established researchers with considerable experience, as well as a number of up-and-coming researchers interested in working in the field. While the links between all these individuals and groups may be many and varied, and several of them may have links with government, it seems that African countries generally have limited fora or processes that make optimal use of existing HTA research capacity. This suggests that efforts to network HTA researchers more closely are required.

*Recommendations for research organisations seeking to strengthen the task network for HTA researchers in their countries or region*

1. Invest considerably in expanding relationships with key stakeholders within the country, region and internationally, but ensure that these relationships are guided by clear objectives. Analysing the nature, strengths and weaknesses of existing networks might be a necessary precursor to this activity.
2. Consider partnering with one or two local organisations on a concrete project in order to enable a larger piece of HTA work, strengthen methodologies and model a collaborative, multi-disciplinary approach to building HTA capacity. Such an activity would also help to cement relationships.
3. Given that there is considerable research expertise in the private sector in some low- and middle-income countries, explore what role this could play in supporting the development of an HTA system. Private sector players could eventually be commissioned to conduct appraisals but these would have to adhere strictly to formal guidelines. It is unclear whether datasets held by the private sector are either suitable or available for use in economic appraisals by independent researchers: this might be one issue that a network could explore.
4. Consider establishing a formal regional network: apart from helping to build capacity regionally, this could also inform the development of priority-setting approaches in the home country. Careful thought would need to be given to how to run and sustain such a network (drawing on international lessons), and how to ensure this network creates meaningful capacity. The involvement of policy-makers would be important for an HTA network and the number of countries involved might need to be limited to enable in-depth discussion and implementation of clearly defined activities. However, it may be premature for research organisations to venture into this domain without prior experience of effective HTA processes in their own country context.

### Strengthening the organisational capacity of HTA research groups

Research groups often find it difficult to develop good organisational capacity (in terms of financial sustainability, sound governance and financial management, skilled researchers and quality assurance). Shifting research groups from a precarious to a more sustainable existence is essential to enable HTA to become a routine component of priority-setting processes.

*Recommendations for strengthening the organisational capacity of research groups*

1. In partnerships with local and international researchers, create structures and processes for decision-making and communication that are mutually satisfying, and actively manage the needs and concerns of the various partners.
2. Incorporate a funding strategy into an organisational capacity-building plan that seeks to move towards long-term and core funding, and put mechanisms in place to preserve academic independence where this funding is under government or donor control.

### Building the capacity of individual research staff to conduct and apply HTAs

While the number of institutions and individual researchers in Africa involved in health economics studies seems to have grown slowly over the past two decades, comprehensive HTA studies are relatively uncommon, which suggests that individual researchers generally do not have the skills and experience for such research.

*Recommendations for research organisations seeking to build the capacity of external and internal research staff*

1. Survey key stakeholders regarding training needs for HTA (in terms of content and format). Also investigate the content and format of existing courses. Finally, investigate the training experience of countries with well-established HTA training programmes. On the basis of this, develop a detailed training strategy.
2. Seek funding and technical support for designing appropriate courses, including the development of locally relevant case studies.
3. Develop a strategy for recruiting local researchers into the organisation, and developing the capacity of all internal staff. On-the-job mentorship of young researchers is important.

It is not essential to send all research staff on formal training programmes overseas. However, there may be fruitful exchanges that can be arranged.

## Conclusions

Research organisations working in the field of HTA need to do more than conduct high quality economic research: they also have to engage with a complex array of stakeholders, network with other research organisations, build partnerships with different levels of government and train the future generation of HTA researchers and policy-makers. In low- and middle-income countries, where there are seldom formal government HTA agencies, they must also support the development of an effective HTA-informed priority-setting process that is contextually sensitive to societal and government needs and priorities. International experience advises that, in embarking on this complex process, it is important to start small, building on existing capacity and opportunities through the development of sound partnerships.

There are clearly many possible dimensions to a capacity-building strategy for HTA in African countries: for each country, the key will be to select appropriate starting points that build on research partners’ strengths, meet some of the immediate needs of the country governments and contribute to longer-term goals.

## Figures and Tables

**Box 1:** Components of an effective and fair HTA-informed priority-setting process

Specific legislation and structures to oversee the use of HTA to inform government decision-making Specific legislation and structures to register new health products that are efficacious and safe to use A systematic process that involves a wide range of stakeholders in identifying policy-relevant interventions for assessment Economic analysis and clinical assessment of priority interventions that use sound methods and are based on criteria that conform to social and health objectives Budget impact analyses that project the financial impact of new interventions A deliberative process that combines the findings of the above analyses with more subjective criteria generated through consultation, and makes recommendations A government decision-making process that assesses these recommendations and decides whether to fund new interventions under the public budget An appeals and evaluation process that allows government decisions to be challenged and assesses the impacts of newly funded interventions A mechanism to adjust and update recommendations on the basis of new information

Sources: [2, 5, 9, 26, 27]

**Box 2:** Lessons for successful networking from three African networks in which the School of Public Health at the University of the Witwatersrand in South Africa was instrumental

Strong support from senior leadership of institutions during start-up Strong, transparent and effective leadership for networks/collaborations Strong anchoring institutions which had the financial and human resources to sustain the network/collaboration, especially at start-up Shared goals and mutual benefits Equal partnerships Clear expectation of active participation Dedicated resources for collaboration in terms of funding and a secretariat Started small and grew subsequently Regular face-to-face meetings Recognised and addressed challenges Refusal to compromise on agreed principles and ethical guidelines (especially in relation to hidden agendas of partners or funders) Supportive administrative systems

Source: Summarised by [2]

## Notes

1. HTAs initially tended to deal with pharmaceuticals, equipment and the like, but have more recently been extended to examining much broader interventions such as public health strategies (the integrated management of childhood diseases, for example) or financing strategies (such as sugar taxes). The term ‘technology’ therefore needs to be understood in its widest sense.

2. These were: ‘Learning from practice: HTA capacity development across Asia,’ hosted by Thailand’s Health Intervention and Technology Assessment Programme (HITAP), the Asia-Pacific Observatory on Health Systems and Policies of the World Health Organisation, and the International Decision Support Network (iDSI); and ‘Priority-Setting for Universal Health Coverage: the role of the International Decision Support Initiative,’ hosted by the UK’s NICE International, Bill and Melinda Gates Foundation, Thailand’s Health Intervention and Technology Assessment Program (HITAP), the Rockefeller Foundation, the UK’s University of York, the USA’s Centre for Global Development, and the Asia-Pacific Observatory on Health Systems and Policies of the World Health Organisation.

3. This workshop was also attended by Thailand’s Health Intervention and Technology Assessment Program (HITAP), the UK’s NICE International and the UK’s University of York which, at the time, had newly joined forces as partners with PRICELESS SA as part of the International Decision Support Initiative (iDSI).

4. The term HTA should possibly be substituted with a more comprehensive and self-explanatory term. Health Intervention and Technology Assessment is the term used in Thailand.

5. This is available at https://www.ispor.org/universal-health-care-coverage_international-decision-support-initiative-reference-case.pdf.

## Paper context

Some experience has been gained by low- and middle-income countries on how to build general capacity in health economics. These experiences are reviewed and lessons drawn on how capacity can be strengthened further in Africa with respect to the specific research areas of economic evaluation, health technology assessment and priority-setting for health. The focus on priority-setting is in response to the urgent need to ensure scarce resources are used effectively in support of Universal Health Coverage, and the scant literature discussing how technical economic evaluations should be integrated into highly political and complex priority-setting processes. Researchers’ roles in developing capacity in these areas are highlighted because few African governments have technology assessment units that can take responsibility for driving formal priority-setting processes.
